# Editorial: Nanotechnology in Traditional Medicines and Natural Products

**DOI:** 10.3389/fchem.2021.633419

**Published:** 2021-02-22

**Authors:** Ruoyu Zhang, Fang Liu, Ye Tian, Wei Cao, Ruibing Wang

**Affiliations:** ^1^ Institute for Chemical Biology and Biosensing, and College of Life Sciences, Qingdao University, Qingdao, China; ^2^ Institute of Tropical Medicine and Artemisinin Research Center, Guangzhou University of Chinese Medicine, Guangzhou, China; ^3^ College of Engineering and Applied Sciences, Nanjing University, Nanjing, China; ^4^ Department of Chemistry, Northwestern University, Evanston, IL, United States; ^5^ State Key Laboratory of Quality Research in Chinese Medicine, Institute of Chinese Medical Sciences, University of Macau, Taipa, China

**Keywords:** natural products, traditional nanomedicines, immunotherapy, drug delivery, artemisnins

**Editorial on the Research Topic**

**Nanotechnology in Traditional Medicines and Natural Products**

As the most important antimalarial drug, artemisinin has gained worldwide attention, and scientists doing pioneering work in related research field have been awarded Nobel Prize in Physiology or Medicine in 2015. As one example of many cases that natural products play important roles in medical and clinical applications, each year, artemisinins save hundreds of thousands of people’s lives around the world. Natural products with medical values include a broad range of substances, which can be obtained directly or extracted from plants, animals, to fungi, yeast, and even minerals. Researchers have spared great efforts to fully exert their potential in promoting health, and preventing and curing diseases. Although with thousands of years of history, traditional medicine remains sort of mysterious and unrevealed due to complex prescription, lack of standard evaluation methods, and effective drug delivery system (Jiang et al., 2010). Even in the well-known example of artemisinin, it takes years’ efforts for scientists to find a proper way to fully utilize its antimalarial activity (Tu, 2016). More profound and systematic research work are demanded to push the development of traditional medicine to a new era. In addition, nanotechnology, which benefits modern medicine by versatile formulation and ease of surface modification, offers promising tools for the development of traditional medicines.

This research topic aims to reflect the current progress in natural products and related nanomedicine. The collection includes bioactivity of natural products, nanofabrication, and delivery methods for targeted and controlled release for cancer treatment. Natural products usually suffer poor water solubility, low stability, and lack of targeting capability. To tackle this problem, various nanocarriers are employed for drug delivery. Nanocarriers such as polymeric NPs, liposomes, and biomacromolecules like DNA have good biocompatibility. In this topic, there are some work and reviews that covers the design and fabrications of nanocarriers for drug delivery (Borcan et al.; Li et al.; Xu et al.). Nanocarriers such as mesoporous silica NPs and carbon nanotubes are good scaffolds for drug loading due to their structural advantages (Ma et al., 2019). It is noteworthy that in some cases, the drug carriers may have the functionalities to suppress the unexpected toxicity of a drug. For example, Gao et al. found that when used for anabasine (ANA) loading, a cucurbituril derivative can significantly inhibit the developmental toxicity of ANA on zebra fish. This work is included in this topic (Gao et al.). In addition, additives are used in nanoformulation to activate or improve the drug efficacy. For instance, research has shown that Fe^2+^ is a very important catalyst for artemisinin activation in Fenton-like reaction that generates free radicals and reactive oxygen species (ROS). The finding also promotes the artemisinin-based chemodynamic therapy (CDT) with the assistance of iron-containing nanoformulation. The latest progresses in this research area have been reviewed by Wu et al. and included in this topic (Wu et al.).

Nanotechnology has also benefited the development of traditional medicine by targeted delivery and controlled release. By attaching ligands such as folic acid, hyaluronic acid, cRGD, transferrin, and aptamers, nanoparticles are prone to accumulate in cancer cells through cell internalization processes, hence help reduce the drug dosage and minimize the side effects on normal tissues (Muhamad et al., 2018; Niu et al.). Cell-derived membranes such as cancer cell membranes provide original complex biological entities that are difficult to replicate artificially. These entities not only help avoid body clearance by immune recognition but also allow specific binding with homotypic tumor cells (Harris et al., 2019). Mesoporous silica nanoparticle loading with quercetin (QT) and wrapping in cancer cell membranes have been reported by Huang et al. and included in this topic for enhanced tumor targeting and radiotherapy, which is a good example that biomimetic nanotechnology helps in natural nanomedicine drug delivery (Huang et al.). Many cancer cells are found to have a lower pH and a higher glutathione level; thus, smart responsive nanoformulation has been designed for drug release at target sites upon stimuli such as pH and redox (Jiang et al.). Versatile formulation technology also allows co-delivery of multiple drugs, since multiple active ingredients are usually employed in traditional medicine. It also facilitates the combination of chemotherapy with photothermo- and photodynamic therapy (Chen et al.). Futher details await your reference in this topic for the nanotechnology in traditional medicines and natural products.

Last decades have witnessed the prosperity of immunotherapy in cancer research, which is fundamentally different from traditional chemotherapy or targeted therapy. It activates hosts’ own immune systems to fight against cancer cells, avoiding exposure of normal cells and tissues directly to the toxicity of drugs. Mounting evidence has shown the great potential of natural products and their derivatives in cancer immunotherapy (Deng et al., 2020). For example, Liu et al. found a water-soluble polysaccharide isolated from *Polyporus umbellatus* Fries, which has significant immune activity and increases the secretion of inflammatory cytokines. The results show that this polysaccharide may be valuable in bladder cancer treatment (Liu et al.). Other typical examples of natural products with anticancer immunologic activity include polyphenols, cardiotonic steroids, and terpenoids (Deng et al., 2020). We hope the abovementioned content included in this topic is useful for exploring the potential of tradition medicine and natural products in immunotherapy in the future.

Drug delivery by nanocarriers has advantages such as targeted delivery, ease of controlled release, higher stability, and delivery efficiency. Moreover, it makes diagnosis and therapy in-one-go possible, offering chances to relieve patients’ pain. Although a wealth of evidence has supported the curative effect of natural product and traditional medicine in disease treatment and cancer therapy, there remain some critical problems and challenges before being widely commercialized. First, most of the research on the nanomedicine of natural product still in the infancy phase of laboratory testing. More work to explore and evaluate their action mechanism and regulation pathway scientifically is needed. In addition, herbal and traditional medicines often adopt complex prescription, which contains more than two active ingredients, making it more difficult to study their curative mechanism. Third, while a plenty of Western nanomedicines such as Doxil, Emend, and Cimzia have been FDA-approved and marketed, there is still a long way to go for traditional medicine and natural products. Standard clinical trials and systematic evaluation are suggested to be established first. More promising results are expected after the merge of the nanomedicine technology and natural products.

## References

[B1] Deng L. J. Qi M. Li N. Lei Y. H. Zhang D. M. Chen J. X. (2020). Natural products and their derivatives: promising modulators of tumor immunotherapy. J. Leukocyte Biol. 108 (2), 493–508. 10.1002/JLB.3MR0320-444R 32678943 PMC7496826

[B2] Harris J. C. Scully M. A. Day E. S. (2019). Cancer cell membrane-coated nanoparticles for cancer management. Cancers 11 (12), 1836. 10.3390/cancers11121836 PMC6966582 31766360

[B3] Jiang Y. David B. Tu P. Barbin Y. (2010). Recent analytical approaches in quality control of traditional Chinese medicines--a review. Anal. Chim. Acta 657 (1), 9–18. 10.1016/j.aca.2009.10.024 19951752

[B4] Ma Z. Fan Y. Wu Y. Kebebe D. Zhang B. Lu P. (2019). Traditional Chinese medicine-combination therapies utilizing nanotechnology-based targeted delivery systems: a new strategy for antitumor treatment. Int. J. Nanomed. 14, 2029–2053. 10.2147/IJN.S197889 PMC6435121 30962686

[B5] Muhamad N. Plengsuriyakarn T. Na-Bangchang K. (2018). Application of active targeting nanoparticle delivery system for chemotherapeutic drugs and traditional/herbal medicines in cancer therapy: a systematic review. Int. J. Nanomed. 13, 3921–3935. 10.2147/IJN.S165210 PMC6038858 30013345

[B6] Tu Y. (2016). Artemisinin-A gift from traditional Chinese medicine to the world (Nobel lecture). Angew Chem. Int. Ed. 55 (35), 10210–10226. 10.1002/anie.201601967 27488942

